# Pigmented Epithelioid Melanocytoma in Congenital Nevus of Medium Size in Children

**DOI:** 10.5826/dpc.1003a67

**Published:** 2020-06-29

**Authors:** Ružica Jurakić Tončić, Slobodna Murat Susic, Danijela Curkovic, Mikela Petkovic, Bostjan Luzar, Ivana Ilic

**Affiliations:** 1 University Hospital Centre Zagreb, Department of Dermatology and Venereology, School of Medicine, University of Zagreb, Croatia; 2 Institute of Pathology, Medical Faculty, University of Ljubljana, Slovenia; 3 University Hospital Centre Zagreb, Department of Pathology and Cytology, University of Applied Health Sciences, Zagreb, Croatia

**Keywords:** melanocytoma, epithelioid pigmented melanocytoma, medium-sized congenital nevus, children

## Introduction

Pigmented epithelioid melanocytoma (PEM) is a very uncommon type of the tumor and, because of its cellularity and atypia, may be mistaken for conventional melanoma [1,2]. PEM clinically presents as a darkly pigmented, slowly growing nodule in young patients [2]. Owing to the dermal infiltrates, it clinically presents with a blue-gray color. Typical localizations are face, trunk, extremities, and genitalia. The term *pigmented epithelioid melanocytoma* was proposed by Zembowicz et al in 2004, who renamed *epithelioid blue nevus*, previously described by Carney [1]. Histologically PEM is characterized by heavily pigmented epithelioid/dendritic cells with prominent nucleoli and pigment located at the periphery of the cytoplasm. Interspersed melanophages are in some lesions prominent and in others rare. Cells show no signs of maturation. Mitoses are infrequent.

The biological behavior is not well known, but generally PEM is considered a low-grade neoplasm [1,2]. Although reports are limited, evidence supports the notion that the tumor follows an indolent clinical course. Of patients who underwent sentinel lymph node biopsies, 60% of patients in the current series and 46% of patients in the series of Zembowicz et al exhibited lymph node metastases, supporting a high rate of regional spread [1]. Only 1 documented case of a distant metastasis of PEM to the liver has been documented. However, the overall survival is excellent, with no reported cases of death.

## Case Presentation

**Figure 1:**
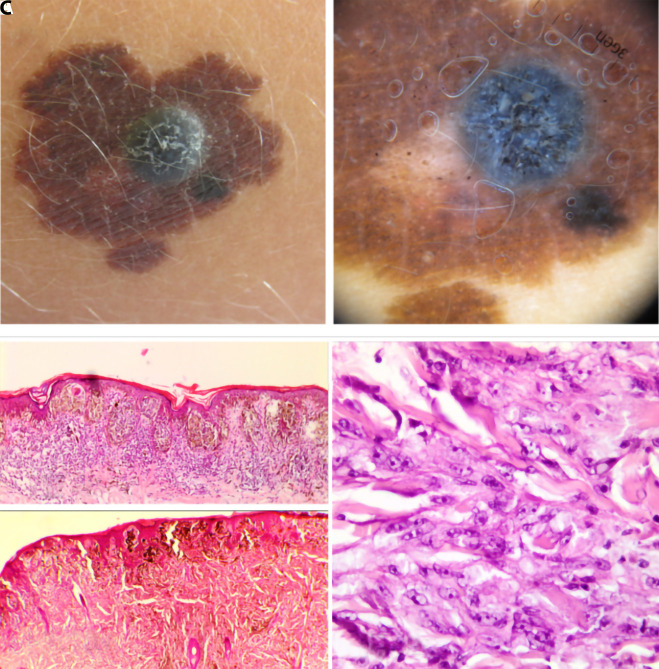
Clinical, dermoscopic, and histological images of the lesion in the first child. (A) Nodular black-blue lesion inside of medium-sized congenital nevus. Camera: Canon PowerShot SX520 HS. (B) Dermoscopy shows metal-blue nodular part and black dots (peppering) around nodular part in surrounding congenital nevus. Photo taken by 3Gen DermLite Foto. (C) In histopathological slides an asymmetrical melanocytic lesion is seen in the epidermis and in a dermis. The epidermal component is made of melanocytic nests of a different size. Most melanocytes in the nests are round and have small nuclei, indistinctive nucleoli, and average-size cytoplasm with some pigment. In a few nests larger melanocytes with larger nuclei and larger nucleoli are seen. The same type of nevoid melanocytes is seen in a dermis admixed with epithelioid and dendritic melanocytes and a large amount of melanophages (H&E, ×4). Dendritic melanocytes show plexiform growth pattern (H&E, ×10; H&E, ×40). Only a few mitoses are seen in epithelioid melanocytes. On the periphery of the lesion is lymphocytic infiltrate.

We present 2 cases of PEM, each of which presented as a newly occurring lesion that was part of a medium-sized congenital nevus. The first child, 3 years of age, presented with rapidly growing nodular changes over 4 months. Urgent excision was made to rule out melanoma in medium-sized congenital nevus. The nevus was excised completely. Histopathological diagnosis of a combined tumor composed of 2 components, PEM and junctional melanocytic nevus, was made in 2 independent expert centers (Figure 1, A–C).

**Figure 2:**
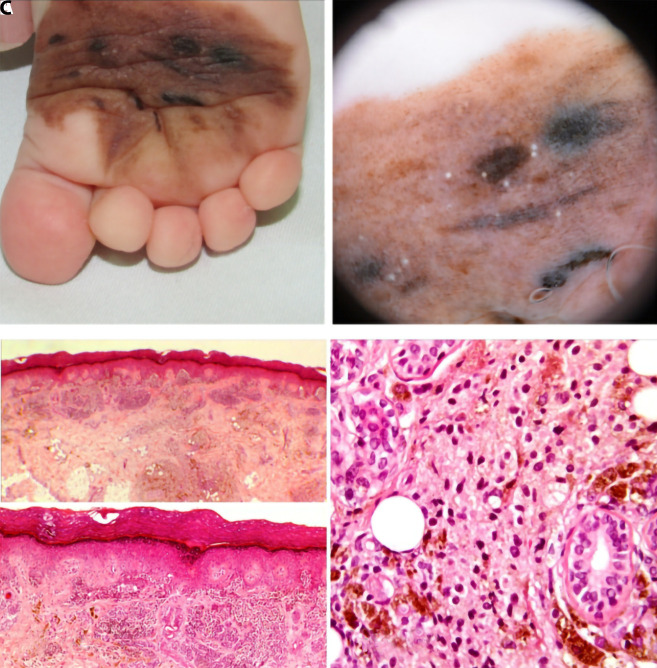
Clinical, dermoscopic, and histological images of the lesion in the second child. (A) Blue-black macule inside the congenital plantar medium-sized nevus. Camera: Canon Powershot SX520 HS. (B) Dermoscopy shows metal-blue part in congenital nevus, with black dots, but less sharply demarcated. Photo taken by 3Gen DermLite Foto. (C) Histologically, the lesion was composed of epidermal and dermal melanocytic nests with the dermal component being partly composed of small oval melanocytes and partly of epithelioid and dendritic melanocytes that grow in a plexiform pattern. There were no mitoses (H&E, ×4; H&E, ×10). There is large amount of melanophages among the melanocytes. The dermal component is partly composed of epithelioid and dendritic melanocytes that grow in a plexiform pattern surrounding the adnexa (H&E, ×40).

The second child, 6 months old, was sent for examination of a medium-sized plantar congenital nevus. The child’s parents noticed that parts of the nevus got darker in comparison with the rest of the nevus. The child was sent for partial excision of the darkest parts of the lesion. The samples were sent for a histopathological analysis and diagnosis of composite melanocytic tumor, made of a congenital type of melanocytic nevus and PEM, was established in 2 independent expert centers (Figure 2, A–C). In both cases, deep penetrating nevus was excluded due to immunohistochemistry.

## Conclusions

Melanocytoma is extremely rare. It was described in another report as a part of the congenital nevus. The size of the nevus was not mentioned, and lesions had been observed since the child’s birth. Our report describes two unusual cases of melanocytoma in medium-sized congenital nevi of children occurring after birth.

The dermoscopic features of PEM were described in a small case series, but no correlation between dermoscopy and histopathology was found [2]. PEM should be considered as low-grade melanocytic tumor with limited metastatic potential, but further studies including follow-up of the patients are needed.
